# Supplementary material for: Intelligent surgical workflow recognition for endoscopic submucosal dissection with real-time animal study
Source: Nat Commun. 2023 Oct 21;14:6676. doi: 10.1038/s41467-023-42451-8 (PMC10590425; doi:10.1038/s41467-023-42451-8)
Supplement: Supplementary file 1 — Supplementary Information [file 41467_2023_42451_MOESM1_ESM.pdf]

Supplementary Information

Intelligent Surgical Workflow Recognition for Endoscopic Submucosal Dissection with Real-time Animal Study

Jianfeng Cao[1], Hon-Chi Yip[2,*], Yueyao Chen[1], Markus Scheppach[3], Xiaobei Luo[4], Hongzheng Yang[1], Ming Kit Cheng[5], Yonghao Long[1], Yueming Jin[6], Philip Wai-Yan Chiu[7,*], Yeung Yam[5,7,8*], Helen Mei-Ling Meng[8,*], Qi Dou[1,*]

[1]Department of Computer Science and Engineering, The Chinese University of Hong Kong, Hong Kong, China
[2]Department of Surgery, The Chinese University of Hong Kong, Hong Kong, China
[3]Internal Medicine III - Gastroenterology, University Hospital of Augsburg, Augsburg, Germany
[4]Guangdong Provincial Key Laboratory of Gastroenterology, Nanfang Hospital, Southern Medical University, Guangzhou, China
[5]Department of Mechanical and Automation Engineering, The Chinese University of Hong Kong, Hong Kong, China
[6]Department of Biomedical Engineering, National University of Singapore, Singapore
[7]Multi-scale Medical Robotics Center and The Chinese University of Hong Kong, Hong Kong, China
[8]Centre for Perceptual and Interactive Intelligence and The Chinese University of Hong Kong, Hong Kong, China

* Corresponding authors: Hon-Chi Yip (hcyip@surgery.cuhk.edu.hk), Philip Wai-Yan Chiu (philipchiu@surgery.cuhk.edu.hk), Yeung Yam (yyam@mae.cuhk.edu.hk), Helen Mei-Ling Meng (hmmeng@se.cuhk.edu.hk), and Qi Dou (qidou@cuhk.edu.hk).

Supplementary Note 1

As a supplement to the annotation protocol in the main text, dataflow and annotation details are provided here for reference.

1). Dataflow of large-scale phase annotation

To simplify the annotation process, we developed a dataflow to inspect the start and end frames (Supplementary Figure 1). Given the large dataset size, we initially reduced the video to 1 frame per second and saved it as a sequence of consecutive images. Subsequently, we created an Excel file to document the phase annotation frame-by-frame. During the annotation process, annotators primarily focused on the downsampled images to identify the start and end frames of each phase (Supplementary Figure 2). They then labeled all frames within this timeframe as corresponding to the designated phase. Additionally, annotators relied on the original video to interpret visual cues that helped differentiate between surgical phases. These cues are crucial in identifying changes in tissue texture, color, or the position of surgical tools because they represent the transition from one phase to the next. For example, during the Marking phase, the endoscopist inspected the contact point between the knife and mucosa. While during the Injection phase, they observed the movement and diffusion of the injected agent inside the tissue. Finally, for the Dissection phase, the annotator looks for changes in tissue color or texture that indicate the separation of different tissue layers. This temporal information is critical for the annotators to confirm whether surgical tools were hovering in the air, denoting the ending of a phase segment.

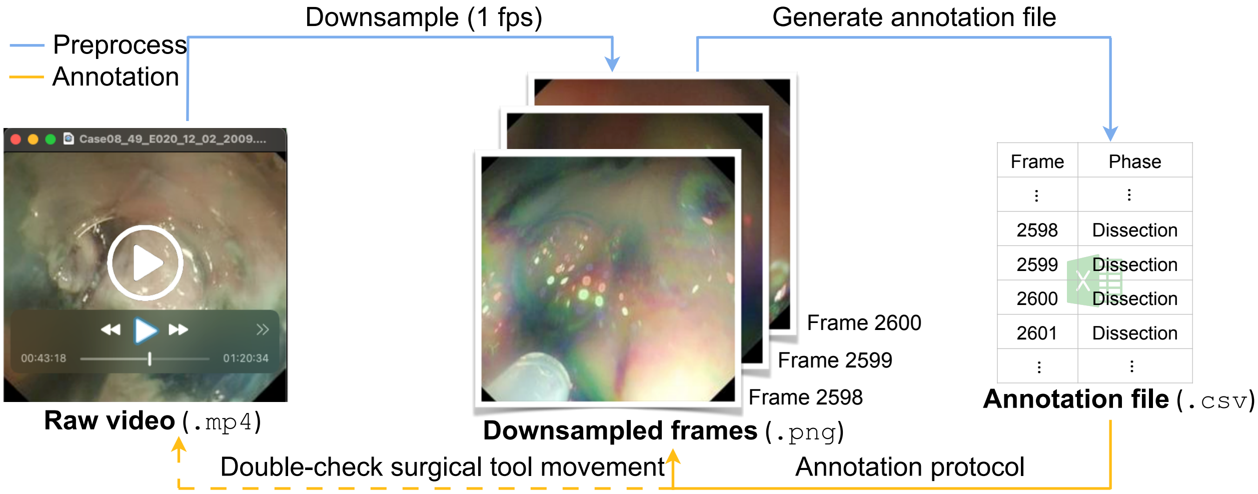

Supplementary Figure 1: The workflow of phase annotation. Blue lines represent the stages we prepared data for annotation, and yellow lines denote the annotation process.

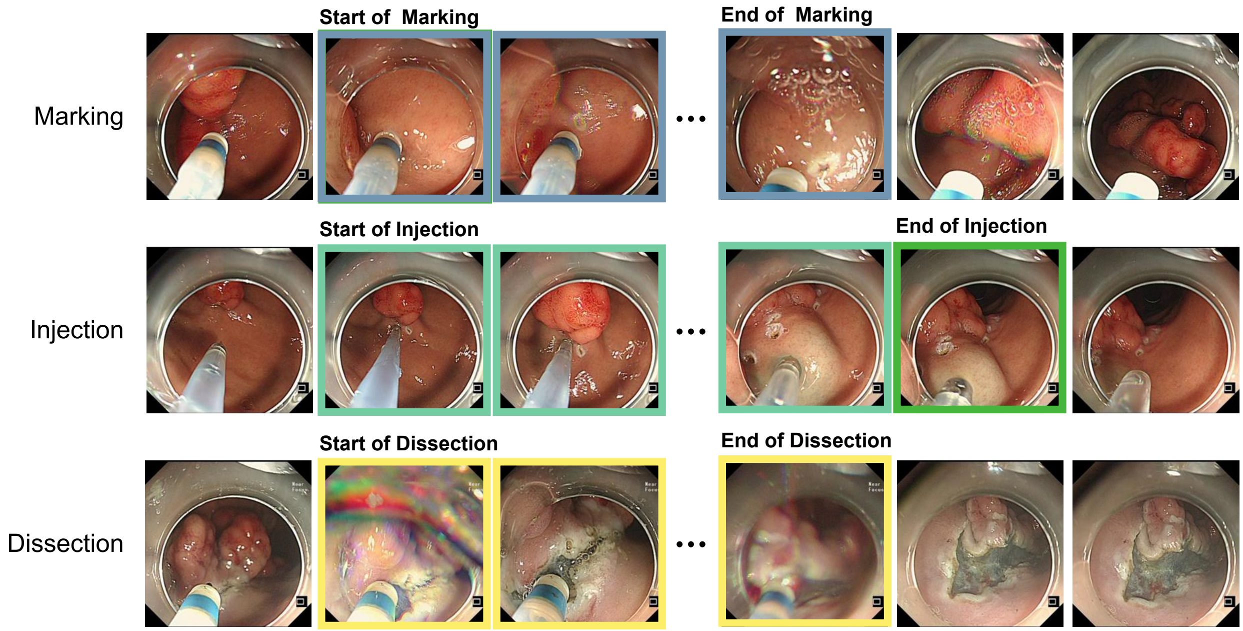

Supplementary Figure 2: The annotation examples of start and end frames of phases Marking, Injection, and Dissection at 1 fps.

---

2). Three-step annotation schedule

Our work adopted a three-step annotation schedule to annotate developmental and external datasets.

The entire annotation process is as follows:

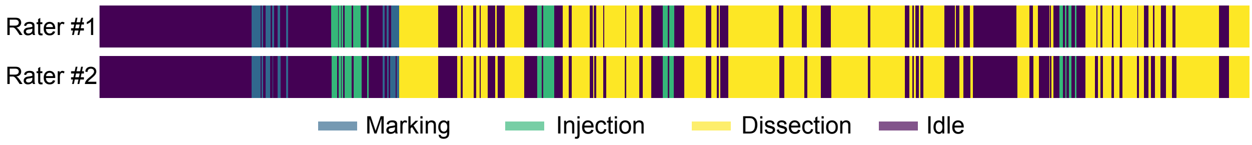

Supplementary Figure 3: Annotation examples of two raters.

(1) To ensure the quality of the annotation process, we first randomly selected approximately 10% (5 cases, 20,446 frames) of developmental dataset and assigned two annotators to individually annotate the data using the dataflow outlined in Supplementary Figure 1, following predefined protocols. This step served as a quality control measure for the annotations, as well as allowing annotators to become familiar with the annotation workflow. To quantitatively evaluate the interobserver agreement between the two raters, we used the Pearson correlation coefficient (PCC) [1]. The resulting PCC for the selected samples was 0.93, indicating a high degree of agreement between raters. To visually examine disagreements between the annotations, we display phase annotation examples of two annotators in Supplementary Figure 3. For the majority of the video, the annotations of the two raters were nearly identical, with only a small percentage (3.30%) exhibiting ambiguity between the annotations of different raters.

(2) Considering the high level of consistency in the initial annotation results of the two annotators, we divided all annotation tasks into roughly equal halves, with each rater individually annotating one part. For instance, in the case of the 47 cases in the expert dataset, we divided the dataset into two non-overlapping parts of 24 and 23 cases, respectively. The two annotators then individually annotated each part, resulting in 108,286 frames for the first part and 92,740 frames for the second part. Given the high level of agreement between the annota-

tors, we treated the two parts of annotations as the final annotation for the expert dataset. We employed the same strategy to distribute the datasets to raters for all external datasets.

(3) Following the completion of all annotation tasks by the two annotators, we subjected the annotations to quality control by two experienced endoscopists with six and three years of experience. Their focus was on correcting challenging annotations that even trained annotators may struggle with. Both endoscopists relied on visual cues and practical surgical experience to determine the appropriate surgical phase in complex surgical sites or when key anatomical landmarks were obscured. To facilitate the verification process, we provided synchronized information by overlaying the annotation results from the second step onto the raw video. The endoscopists reviewed the video and corrected any errors as needed. In the case of any discrepancies, the two experienced endoscopists discussed for a consensus label, while with more respect to the relatively senior one who has conducted more ESD cases. This step yielded the final phase annotation dataset.

Supplementary Note 2

The orderliness metric is derived from the Youden index's optimal cut-off and measures the separability between the target phase and the other phases. We named this metric orderliness to differentiate it from the commonly used accuracy metric, which employs a fixed threshold of 0.5. The optimal threshold of the Youden index is determined by maximizing the summarization of sensitivity and specificity [2], resulting in a more accurate measurement of how well the positive and negative samples are separated relative to the optimal threshold on the probability scale bar [0, 1]. The Youden index has been extensively discussed in previous research as a means of characterizing the class-wise performance of a model on a multi-class classification problem [3, 4].

The model outputs the probability of each frame belonging to one of four distinct phases: Marking, Injection, Dissection, and Idle. This allows us to evaluate the model's performance both overall and for each individual phase. To help clarify the phase-wise metric orderliness presented in Figure 2b, we will use the example of the Dissection phase to illustrate how orderliness captures the order of frames within a phase. We begin by taking the probability of each frame x being Dissection, denoted as $p(\text{Dissection}|x)$. Using the `sklearn.metrics.roc_curve` package, we generated a ROC curve that plots the true positive rate (TPR) against the false positive rate (FPR) for thresholds uniformly sampled in the range [0, 1]. We then calculated the optimal threshold $t^* = 0.4$ from the Youden Index, $J_{max} = \max_t\{sensitivity + specificity - 1\} = TPR + (1 - FPR) - 1 = TPR - FPR$. With the optimal threshold t^*, we divided the samples into four groups: $\hat{TP}, \hat{TN}, \hat{FP}$, and $\hat{FN}$. Finally, we defined $orderliness = \frac{\hat{TP}+\hat{TN}}{\hat{TP}+\hat{TN}+\hat{FP}+\hat{FN}}$ to measure the proportion of frames that were correctly sorted relative to the threshold t^*.

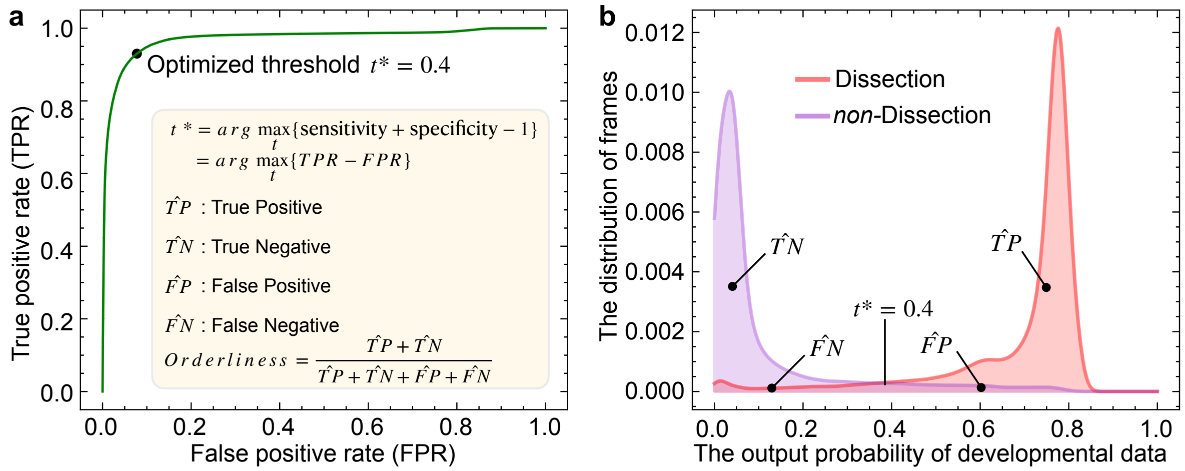

Supplementary Figure 4: Definition of metrics Orderliness. **a** The steps for calculating Orderliness; **b** The distributions of output probabilities corresponding to Dissection (in red) and non-Dissection (in purple) frames.

We have visualized the steps involved in calculating the orderliness metric of phase Dissection in Supplementary Figure 4. In Supplementary Figure 4a, we obtained the optimized threshold t^* from the ROC, and the inset illustrates the specific process we used to calculate orderliness, as described above. Additionally, we have depicted the relationship between the threshold t^* and the probability distributions of Dissection and non-Dissection frames in Supplementary Figure 4b. Ideally, t^* would be able to perfectly discriminate between the boundaries of these two distributions, which means the output probability of Dissection frames should be higher than that of non-Dissection frames. However the AI model may fail to recognize challenging scenarios, such as a blurry view or obscured surgical tools. This can result in prediction errors, i.e., $\hat{FP}$ and $\hat{FN}$, consequently dividing all samples into four groups, as shown in the figure. To assess the performance of the AI model with respect to each phase, we define the *orderliness* to calculate the proportion of frames that are correctly classified, i.e., $\hat{TP}$ and $\hat{TN}$.

Supplementary Table 1: Statistical analysis on the diversity of developmental dataset.

**Group**	**Varity**	**Percentage**
Exception	Bleeding	25.53%
	Rectum	6.38%
Location	Stomach	87.23%
	Esophagus	6.38%
	2008∼2012	25.53%
Date	2012∼2016	38.30%
	2016∼2020	36.17%
	Dual knife	72.34%
Dissection tool	Isolation-tipped knife	10.64%
	Triangle-tipped knife	17.02%

Supplementary Table 2: Performance metrics on external dataset from different surgeons and skills. Source data are provided as a Source Data file.

**Phase-wise metric**	**AUROC**	**Specificity**	**Sensitivity**	**Orderliness**
Marking	97.30 (94.45, 100.0)	93.23 (88.35, 98.12)	94.09 (89.79, 98.40)	93.23 (88.36, 98.11)
Injection	98.87 (98.06, 99.68)	96.53 (95.19, 97.87)	96.69 (95.16, 98.23)	96.53 (95.20, 97.86)
Dissection	95.69 (92.34, 99.05)	93.10 (91.01, 95.19)	90.16 (85.96, 94.36)	92.36 (90.47, 94.24)
Idle	96.68 (95.82, 97.55)	90.82 (88.55, 93.09)	92.07 (90.41, 93.73)	91.67 (90.11, 93.22)
Average	97.14 (95.17, 99.07)	93.42 (90.78, 96.07)	93.25 (90.33, 96.18)	93.20 (91.04, 95.86)

Supplementary Table 3: Performance metrics on ex-vivo animal trial dataset. Source data are provided as a Source Data file.

**Phase-wise metric**	**AUROC**	**Specificity**	**Sensitivity**	**Orderliness**
Marking	57.87 (0.000, 100.0)	35.65 (0.000, 100.0)	86.17 (53.94, 100.0)	36.23 (0.000, 100.0)
Injection	96.97 (92.16, 100.0)	94.11 (80.66, 100.0)	96.44 (91.64, 100.0)	94.14 (81.01, 100.0)
Dissection	95.67 (90.65, 100.0)	89.32 (82.48, 96.15)	90.81 (83.64, 97.98)	90.34 (83.47, 97.21)
Idle	94.74 (88.68, 100.0)	88.13 (78.03, 98.22)	90.13 (84.09, 96.18)	89.08 (81.19, 96.96)
Average	86.31 (67.87, 100.0)	76.80 (60.29, 98.34)	90.89 (78.33, 98.54)	77.45 (61.42, 98.54)

Supplementary Table 4: Performance metrics on in-vivo animal trial dataset. Source data are provided as a source data file.

**Phase-wise metric**	**AUROC**	**Specificity**	**Sensitivity**	**Orderliness**
Marking	69.02 (56.35, 81.69)	53.98 (39.08, 68.89)	90.58 (83.52, 97.64)	54.61 (39.93, 69.29)
Injection	96.58 (95.03, 98.12)	91.64 (89.11, 94.17)	91.82 (86.51, 97.13)	91.72 (89.43, 94.00)
Dissection	94.93 (92.97, 96.89)	91.57 (89.89, 93.24)	86.68 (83.22, 90.14)	88.95 (87.11, 90.79)
Idle	93.27 (92.12, 94.43)	81.62 (78.62, 84.63)	91.24 (89.75, 92.72)	85.47 (83.63, 87.31)
Average	88.45 (84.12, 92.78)	79.70 (74.18, 85.23)	90.08 (85.75, 94.41)	80.19 (75.03, 85.35)

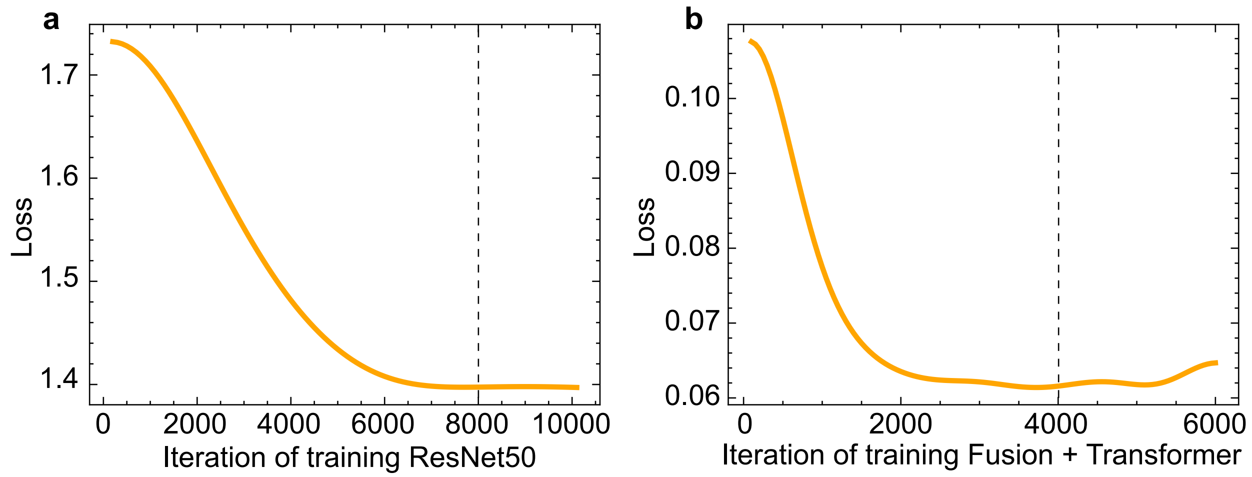

Supplementary Figure 5: The curves of the training loss of (**a**) ResNet50 module in the 1st stage and (**b**) Fusion and Transformer modules in the 2nd stage. The dashed line indicates the number of iterations we actually trained with.

Supplementary References

[1] Hyuk Soon Choi and Hoon Jai Chun. “Accessory devices frequently used for endoscopic submucosal dissection”. In: *Clinical endoscopy* 50.3 (2017), pp. 224–233.

[2] David J Hand and Robert J Till. “A simple generalisation of the area under the ROC curve for multiple class classification problems”. In: *Machine learning* 45 (2001), pp. 171–186.

[3] Miguel De Figueiredo, Christophe BY Cordella, Delphine Jouan-Rimbaud Bouveresse, Xavier Archer, Jean-Marc Bégué, and Douglas N Rutledge. “A variable selection method for multiclass classification problems using two-class ROC analysis”. In: *Chemometrics and Intelligent Laboratory Systems* 177 (2018), pp. 35–46.

[4] Christos T Nakas, Todd A Alonzo, and Constantin T Yiannoutsos. “Accuracy and cut-off point selection in three-class classification problems using a generalization of the Youden index”. In: *Statistics in medicine* 29.28 (2010), pp. 2946–2955.
