# Supplementary material for: Intelligent surgical workflow recognition for endoscopic submucosal dissection with real-time animal study
Source: Nat Commun. 2023 Oct 21;14:6676. doi: 10.1038/s41467-023-42451-8 (PMC10590425; doi:10.1038/s41467-023-42451-8)
Supplement: Supplementary file 3 — Reporting Summary [file 41467_2023_42451_MOESM3_ESM.pdf]

nature portfolio

Corresponding author(s): Hon-Chi Yip, Philip Wai-Yan Chiu, Yeung Yam, Helen Mei-Ling Meng, Qi Dou

Last updated by author(s): Oct 5, 2023

Reporting Summary

Nature Portfolio wishes to improve the reproducibility of the work that we publish. This form provides structure for consistency and transparency in reporting. For further information on Nature Portfolio policies, see our Editorial Policies and the Editorial Policy Checklist.

Statistics

For all statistical analyses, confirm that the following items are present in the figure legend, table legend, main text, or Methods section.

n/a	Confirmed	
☐	☒	The exact sample size (*n*) for each experimental group/condition, given as a discrete number and unit of measurement
☐	☒	A statement on whether measurements were taken from distinct samples or whether the same sample was measured repeatedly
☐	☒	The statistical test(s) used AND whether they are one- or two-sided<br>*Only common tests should be described solely by name; describe more complex techniques in the Methods section.*
☒	☐	A description of all covariates tested
☒	☐	A description of any assumptions or corrections, such as tests of normality and adjustment for multiple comparisons
☐	☒	A full description of the statistical parameters including central tendency (e.g. means) or other basic estimates (e.g. regression coefficient) AND variation (e.g. standard deviation) or associated estimates of uncertainty (e.g. confidence intervals)
☐	☒	For null hypothesis testing, the test statistic (e.g. *F*, *t*, *r*) with confidence intervals, effect sizes, degrees of freedom and *P* value noted<br>*Give P values as exact values whenever suitable.*
☒	☐	For Bayesian analysis, information on the choice of priors and Markov chain Monte Carlo settings
☒	☐	For hierarchical and complex designs, identification of the appropriate level for tests and full reporting of outcomes
☐	☒	Estimates of effect sizes (e.g. Cohen's *d*, Pearson's *r*), indicating how they were calculated

Our web collection on statistics for biologists contains articles on many of the points above.

Software and code

Policy information about availability of computer code

Data collection	Olympus endoscopy system was used for collecting developmental dataset, animal trial cases and part of external dataset (15 external cases conducted with different surgeons and skills, 4 cases with pocket creation and line-assisted traction techniques and 4 cases from Internal Medicine III – Gastroenerology, University Hospital of Augsburg, Augsburg, Germany); Fujifilm endoscopy system was used to record the 4 cases from Nanfang Hospital, Southern Medical University, Guangzhou, China.
Data analysis	All graphical and statistical analyses were performed using python 3.6.13 and pytorch 1.10.2. Code implementation can be available at https://zenodo.org/badge/latestdoi/665438538

For manuscripts utilizing custom algorithms or software that are central to the research but not yet described in published literature, software must be made available to editors and reviewers. We strongly encourage code deposition in a community repository (e.g. GitHub). See the Nature Portfolio guidelines for submitting code & software for further information.

Data

Policy information about availability of data

All manuscripts must include a data availability statement. This statement should provide the following information, where applicable:

- Accession codes, unique identifiers, or web links for publicly available datasets
- A description of any restrictions on data availability
- For clinical datasets or third party data, please ensure that the statement adheres to our policy

The ex-vivo and in-vivo animal trial data used in this study are publicly available at https://doi.org/10.6084/m9.figshare.23506866.v5. Due to ethical regulations on confidentiality and privacy, access to the human cases in the developmental and external datasets of this study is limited to authorized researchers approved by the ethics committee. Source data are provided with the manuscript.

Research involving human participants, their data, or biological material

Policy information about studies with human participants or human data. See also policy information about sex, gender (identity/presentation), and sexual orientation and race, ethnicity and racism.

Reporting on sex and gender	The human data used on development and validation of the artificial intelligence system were de-identified without any personal information. Only endoscopy system information, endoscopic view, surgeon name, and tumor location were preserved for retrospective study. The sex and gender information were not included in the database.
Reporting on race, ethnicity, or other socially relevant groupings	The human data used on development and validation of the AI system were de-identified without any personal information. The race / ethnicity information were not included in the study.
Population characteristics	All these information were de-identified during development and validation of the AI system.
Recruitment	Recorded ESD videos performed were retrospectively collected and no patient identification were used throughout the study.
Ethics oversight	The study received animal study ethics approval from Ethics Committee of The Chinese University of Hong Kong (No. 22-145-MIS).

Note that full information on the approval of the study protocol must also be provided in the manuscript.

Field-specific reporting

Please select the one below that is the best fit for your research. If you are not sure, read the appropriate sections before making your selection.

☒ Life sciences ☐ Behavioural & social sciences ☐ Ecological, evolutionary & environmental sciences

For a reference copy of the document with all sections, see nature.com/documents/nr-reporting-summary-flat.pdf

Life sciences study design

All studies must disclose on these points even when the disclosure is negative.

Sample size	The data used in this study includes developmental dataset (47 cases from Prince of Wales Hospital, Hong Kong), validation dataset on cases with different surgeons and skills (15 cases from Prince of Wales Hospital, Hong Kong), validation dataset on, ex-vivo dataset (4 cases from Prince of Wales Hospital, Hong Kong), in-vivo dataset (12 cases from, Prince of Wales Hospital, Hong Kong), and multi-center datasets (4 cases from Internal Medicine III – Gastroenterology, University Hospital of Augsburg, Augsburg and 4 cases from Nanfang Hospital, Southern Medical University in Guangzhou, China). The number of cases in each dataset is determined according to cases available at each clinical center. For the developmental dataset, we kept the completeness and variety of collected cases. The collected datasets are sufficient for training the model which achieves an average accuracy higher than 90% on the developmental dataset. For the others valiation dataets, we selected cases from different centers and endoscopy systems, which are sufficient for validating the robustness and performance of AI-Endo under complex application scenarios.
Data exclusions	Only complete ESD videos of colorectum, stomach, and esophagus were included in this works. The cases with incomplete recordings or on other organs were excluded.
Replication	Our attempts at reproducibility involved analyzing multiple groups of cases from different endoscopists, endoscopy systems, hospitals and application scenarios. In this work, the proposed phase recognition system AI-Endo retained high performance on expert dataset (Figure 2b), ex-vivo animal trial (Supplementary Table 2), in-vivo animal trial (Supplmentary Table 3), and multi-center dataset (Figure 6). Besides, the source code and animal trial datasets are provided as open source. Therefore, the results presented in this work are supposed to keep high replicability.
Randomization	In the validation of AI-Endo on different surgeons and surgical skill analysis, the cases are grouped according to the analysis target. Specifically, in section "Performance of AI-Endo model on external datasets", 15 cases are grouped into surgeon A (10 cases), surgeon B (2 cases), and surgeon C (3 cases). To analyze the difference of Normalized Transition index, 12 samples were grouped into 5 cases of

	experienced surgeon and 7 cases of novice surgeon, respectively. The sample cases are allocated according to the endoscopist's name who conducted the operation.
Blinding	All analysis in this study were objective and involved the application of mathematical equations to the results of phase recognition, and the trained AI model will not be influenced by participant or researcher bias. Therefore, blinding method is not used in this study. .

Reporting for specific materials, systems and methods

We require information from authors about some types of materials, experimental systems and methods used in many studies. Here, indicate whether each material, system or method listed is relevant to your study. If you are not sure if a list item applies to your research, read the appropriate section before selecting a response.

Materials & experimental systems

n/a	Involved in the study
☒	☐ Antibodies
☒	☐ Eukaryotic cell lines
☒	☐ Palaeontology and archaeology
☐	☒ Animals and other organisms
☐	☒ Clinical data
☒	☐ Dual use research of concern
☒	☐ Plants

Methods

n/a	Involved in the study
☒	☐ ChIP-seq
☒	☐ Flow cytometry
☒	☐ MRI-based neuroimaging

Animals and other research organisms

Policy information about studies involving animals; ARRIVE guidelines recommended for reporting animal research, and Sex and Gender in Research

Laboratory animals	A total of two female pigs (large white breed, weights ~ 30 kg) were used in this work. The age of pigs were not used as the criterion for selection.
Wild animals	No wild animals were used in the study.
Reporting on sex	Because only video content was used for developing AI model for endoscopic scene understanding, the performance of AI model is independent of sex. Therefore, data was not reported disaggregated for sex .
Field-collected samples	No field-collected samples were used in this study.
Ethics oversight	Ethical approvals were obtained from the Ethics Committee of The Chinese University of Hong Kong (No. 22-145-MIS).

Note that full information on the approval of the study protocol must also be provided in the manuscript.

Clinical data

Policy information about clinical studies

All manuscripts should comply with the ICMJE guidelines for publication of clinical research and a completed CONSORT checklist must be included with all submissions.

Clinical trial registration	Not applicable for retrospective study on endoscopic recordings.
Study protocol	Intelligent algorithms were used to processing ESD videos, which are all de-identified.
Data collection	ESD cases were collected from Prince of Wales Hospital, Hong Kong (66 cases); Nanfang Hospital, Southern Medical University in Guangzhou, China (4 cases); and Internal Medicine III – Gastroenerology, University Hospital of Augsburg, Augsburg, Germany (4 cases). All patient information are excluded from the dataset. Only recording information, such as resolution, imaging system and surgeon name, are preserved for statistical analysis on AI-Endo system. Patient's personal information were de-identified. The IRB from Ethics Committee of The Chinese University of Hong Kong has approved that consent is not required from patients.
Outcomes	An automatic phase recognition system AI-Endo was developed to recognize surgical phase of endoscopic video in real-time.
